# Transplant Glomerulopathy: The Interaction of HLA Antibodies and Endothelium

**DOI:** 10.1155/2014/549315

**Published:** 2014-03-09

**Authors:** William Hanf, Claudine S. Bonder, P. Toby H. Coates

**Affiliations:** ^1^Central Northern Adelaide Renal & Transplantation Service, Royal Adelaide Hospital, Adelaide, SA 5000, Australia; ^2^Centre for Cancer Biology, SA Pathology, Frome Road, Adelaide, SA 5000, Australia; ^3^Department of Medicine, University of Adelaide, Frome Road, Adelaide, SA 5000, Australia; ^4^Centre for Stem Cell Research, University of Adelaide, Frome Road, Adelaide, SA 5000, Australia; ^5^Clinical and Experimental Transplantation Group, Royal Adelaide Hospital, North Terrace, Adelaide, SA 5000, Australia

## Abstract

Transplant glomerulopathy (TG) is a major cause of chronic graft dysfunction without effective therapy. Although the histological definition of TG is well characterized, the pathophysiological pathways leading to TG development are still poorly understood. Electron microscopy suggests an earlier appearance of TG and suggests that endothelial cell injury is the first sign of the disease. The pathogenic role of human leukocyte antigen (HLA) antibodies in endothelial cells has been described in acute vascular and humoral rejection. However the mechanisms and pathways of endothelial cell injury by HLA antibodies remain unclear. Despite the description of different causes of the morphological lesion of TG (hepatitis, thrombotic microangiopathy), the strong link between TG and chronic antibody mediated rejection suggests a major role for HLA antibodies in TG formation. In this review, we describe the effect of classes I or II HLA-antibodies in TG and especially the implication of donor specific antibodies (DSA). We update recent studies about endothelial cells and try to explain the different signals and intracellular pathways involved in the progression of TG.

## 1. Introduction

Since the 1970s, kidney transplantation has served as the strong hold to cure chronic kidney disease. However more than 50% of transplant recipients experience late allograft rejection after 5 to 10 years which presents as a significant clinical problem and remains a major barrier to maximizing the utility of transplanted kidneys. Recurrent primary disease, toxicity of immunosuppressive therapy, and late renal rejection all contribute to late transplant loss and significantly reduce the transplant half-life. Whilst acute antibody mediated rejection (AMR) is well recognised as an early cause of graft dysfunction, the chronic late lesion of AMR is less well studied and therapeutic strategies to treat this entity are lacking. With the improvement in management of acute rejection and acute rejection rates now being less than 15% in many centres, management of chronic antibody mediated rejection and its final pathological entity transplant glomerulopathy (TG) has become a major unmet need of transplant nephrology, for which new treatment strategies are urgently required. Prior to 2005 the term “chronic allograft nephropathy” was used to cover a variety of pathological lesions without specific cause. Transplant glomerulopathy itself is a form of chronic allograft nephropathy with poor graft outcomes and a distinctive pathological appearance [1–5]. However, a recent study showed different outcomes between these 2 entities [6].

The pathological features of TG include a multilamination and double contour formation of glomerular basement membrane (GBM) in the absence of immune-complex deposit and are identifiable by Periodic Acid Schiff or silver staining using light microscopy.

Patients with a TG histological diagnosis present frequently with a nephrotic range proteinuria and/or hypertension and/or kidney graft function deterioration as illustrated in Table 1. Peritubular capillary C4d staining has also been considered recently for the diagnosis of antibody mediated kidney rejection but is not correlated well with TG. Interestingly, study using electron microscopy showed early modification of endothelial cells (EC) suggesting earlier appearance of TG [6–10].

The new concept EC injury predefining TG raises the question about the possible crosstalk between these cells and HLA antibodies [11]. Despite different medical strategies (based on acute humoral rejection treatment strategies) to treat TG, none of them appear effective [12]. Some trials with new drugs like eculizumab or bortezomib are ongoing and suggest a noncomplement pathway in TG [13].

This review will cover the morphology and clinical outcomes of TG, the role of HLA antibodies, and a focus on EC injury as a key concept in the TG process.

## 2. Definition of Transplant Glomerulopathy (TG)

**Figure 1:**
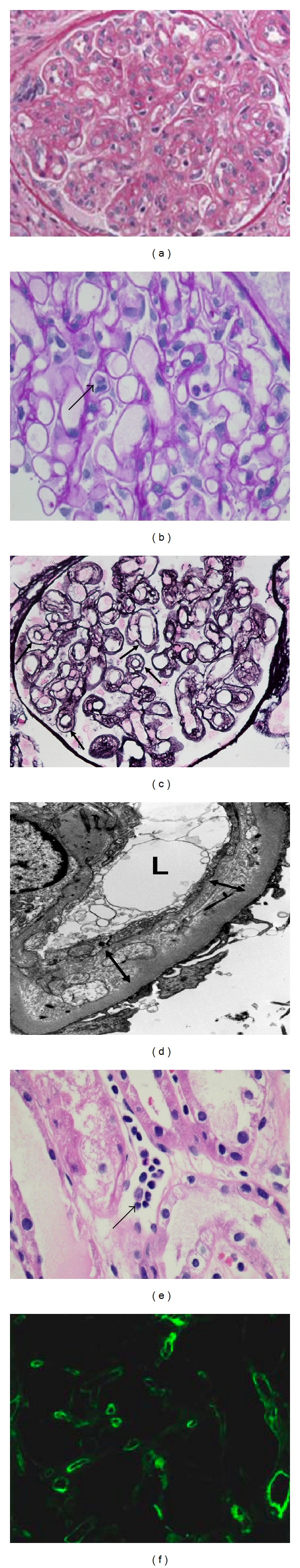
Histopathology of transplant glomerulopathy. (a) Light microscopy showing TG with a glomeruli showing sclerosis (periodic-acid-Schiff stain ×400). (b) Light microscopy showing glomerulitis (presence of mononuclear cells in glomerular capillaries) in a biopsy specimen (periodic-acid-Schiff stain ×600) (c) Silver staining highlighting double contouring of the glomerular capillary wall (×400). (d) Electron microscopy showing duplication of the glomerular basement membrane—arrows (×8000) (e) Peritubular capillitis (arrow) in a TG biopsy specimen (periodic-acid-Schiff ×600) (f) Peritubular C4d staining in a TG biopsy (immunofluorescence ×400).

TG was first recognised in the early 1980s with characteristic features defined as mesangial and EC changes in transplantation kidney graft biopsies [14]. The cardinal features observed in biopsy series included (i) light microscopy identifying a duplication of glomerular basement membrane (GBM), mesangial matrix expansion, and glomerulitis, (ii) electron microscopy (EM) identifying a loss of endothelial fenestration, endothelial cell swelling, and mesangial matrix expansion, and (iii) immunofluorescence identifying mesangial IgM and C3 staining ± C4d in glomerular cells and peritubular capillaries [15, 16]. The manifestations of all these changes are only observed in the lesions of advanced TG but are frequently absent in the early TG lesion. Basement membrane lamellation, which is the earliest sign of TG can be detected by EM within the first 3 months posttransplantation [8] which supports use of early protocol biopsies to survey the allograft to predict its decline in function [17].  Figure 1 summarizes histological lesions observed in TG. Being associate with these light and electronic microscopic lesions, our group frequently observed the presence of PCT inflammation which raises the question about the earliest target in TG. It is tantalising to predict that EC are the first target of HLA class I Ab as they are present in the surface of glomerular and juxtaposed to the PCT cells. Is it possible that lymphocytes use the same process to aggress the glomerular and/or PCT ECs via a possible link with HLA Ab and thereby increases the risk of TG development?

Despite TG being recognised for more than 30 years ago, the mechanisms involved in the development of TG remain largely unknown. Other histological appearances in graft recipients (e.g., thrombopathic microangiopathy, membranoproliferative glomerulonephritis, or lupus nephritis) make the diagnosis difficult [18].

## 3. TG Risk Factors and Clinical Outcome

The development of TG is associated with poor kidney graft outcomes [1–5]. A number of associated factors for the development of TG have been identified of the recipients including age, presence of antibodies directed towards HLA molecules especially class II donor specific antibodies (DSA), C hepatitis positive serology (HCV), and last acute rejection beyond 3 months post-Tx [9, 19]. Unfortunately de novo DSA appearance has been identified in younger recipients and attributed to their noncompliance to immunosuppressive therapy [20]. De novo DSA after transplant result in the increase of chronic humoral injury with development of TG [21].

Gloor et al. showed also that subclinical TG detected in protocol biopsies affects long-term graft outcomes [19]. Our previous study showed that allografts presenting with TG 10 years after transplantation experienced a 33% reduction in graft survival versus 63% in the matched control group [22]. This has been confirmed in other reports [4, 10]. It has been observed that around 4% and 20% of transplant patients present with TG at 1 and 5 years, respectively, after transplantation [19] but some studies showed a lower cumulative incidence of TG [9, 10, 16, 23]. In a previous 16-year retrospective registry analysis we observed a TG diagnosis in 4% of patients [22]. The true frequency of TG is probably underestimated because many centers do not perform protocol systematic biopsy and likely miss the subclinical TG [24]. The mean duration from transplant to diagnosis of TG is 2 to 9 years in clinically indicated biopsies [9, 19, 22, 25, 26]. The main presentation of the earlier stage of clinical TG is the late appearance of subnephrotic range proteinuria [9, 10]. In some cases it is associated with hypertension and ultimately with decline renal function as a late and inevitable complication. A recent study confirms that the appearance of proteinuria one year after transplantation without evidence of TG may predict the development of TG 5 years after transplantation in sensitized patients [4].

## 4. HLA Antibody and Association with TG

It is well accepted that HLA DSA are associated with poor kidney allograft outcomes and that their presence is strongly associated with the development of TG. Preformed DSA, which are present at the time of transplant, persisting after transplantation despite desensitization therapy or de novo DSA are considered a risk factor contributing to TG [27, 28].

We previously showed that 50% of patients presenting with TG had DSA and poor graft survival [22]. Moreover it is well documented that TG is associated with humoral rejection according to the Banff criteria [29, 30]. The presence of HLA antibodies and/or the presence of peritubular capillary inflammation were/was often associated with GBM thickening [30]. The term of chronic allograft nephropathy disappeared after the 2005 Banff meeting [31]; TG term still remains applicable with an overlapping between TG and chronic humoral rejection [26] in part mediated by DSA. More recently a French study confirmed the association of TG anti-HLA antibodies and a new defined antibody-mediated vascular rejection in kidney allograft [32]. The second part of chronic humoral rejection diagnosis is based on peritubular capillary (PTC) C4d deposits [29] similar to the acute humoral rejection criteria [31].

## 5. C4d Staining in Transplant Glomerulopathy

The demonstration of antibody deposition in the graft is an important component of making a diagnosis of antibody mediated allograft injury. The last Banff working group meeting revised in 2011 the definition of antibody mediated rejection (AMR) and acknowledged that the chronic humoral rejection and/or TG development could be C4d negative [7, 33]. PCT C4d staining biopsies is strongly associated with DSA in TG biopsies [19, 26]. Glomerular C4d may occur without C4d staining in PTC in recipients who frequently presented with DSA [9, 19, 34, 35]. Glomerular C4d deposition is a useful additional marker for making the diagnosis of TG and has been proposed as an index of severity in TG. Indeed Batal et al. observed significant higher chronic glomerular lesion score (cg) in paraffin-embedded biopsies that were associated with positive glomerular basement membrane staining as well as a trend toward a significant difference in frozen specimens with glomerular staining [36]. The interpretation of glomerular C4d staining is further complicated by the choice of tissue in which it is detected. Normal glomeruli examined by immunofluorescence in frozen section may show peripheral C4d staining and therefore use of paraffin section is preferred when seeking evidence of glomerular deposition [37]. Loupy et al. described different stages of TG mediated by preformed DSA according to early kidney graft biopsies [27]. The remaining question about the DSA threshold and subsequent development of TG is still unclear and needs to be addressed with further studies including patients with DSA after transplantation [38]. Despite its usefulness when present, C4d staining can be problematic in that its detection varies depending on biopsy series and technique employed for detection [37]. Chronic antibody mediated injury likely occurs in wave like patterns, with variable antibody production, and therefore biopsy may potentially miss periods of peak antibody production and therefore C4d positivity. These pathogenic variations in disease activity and variation in technical detection techniques therefore largely account for the range of C4d positivity reported in biopsy series, which ranges between 25–61% of TG cases described in the literature. Avoiding overdependence on C4d as a diagnostic cornerstone of antibody-mediated rejection and taking other factors into consideration, for example, presence of DSA and other histological evidence of TG (glomerulitis/PTCitis), are likely to contribute to better diagnosis in the future, but this will require further studies to confirm the utility of these factors. Recently the concept of C4d negative antibody mediated rejection has been proposed, which recognises that C4d staining is problematic and proposes a new classification which includes evidence of allograft injury based on light microscopic features of glomerulitis (Banff “g” lesions) and peritubular capillitis (“ptc” lesions). This new classification from the Banff 2013 meeting has not yet been published and validated but it may alter the diagnostic criteria for antibody mediated rejection. Finally, newer techniques including microarray analysis of TG biopsies may provide molecular signals and intracellular pathway activation to allow the diagnosis of TG without histological controversy and may potentially identify new pathways that can be targeted in intervention strategies.

## 6. HLA Antibody Specificity and TG 

Table 1 summarized the percentage of class I and class II HLA Abs in the different studies including TG patients. The link between anti-HLA DSA and TG is well recognised. Moreover, studies report the main risk factor of TG being DSA HLA class II antibodies. However HLA class I antibodies have also been identified [21]. A recent study demonstrated a correlation between HLA class II Ab in de novo DSA after transplantation and poor graft survival [20]. However complete dominance of HLA class II Ab in TG is unlikely even if some explanation has been proposed [20, 26, 39–41]. The important question regarding the risk of TG according to the time of DSA appearance needs to be addressed. For example, are pretransplant DSA as deleterious as posttransplantation DSA for TG lesion development? The majority of studies on TG focused on DSA after transplantation [6, 9, 21] and in fact excluded patients with DSA pretransplantation [19, 20]. We observed that pretransplant DSA were a risk factor of TG but statistical analyses were not performed according to the small number of TG patients. We also described a higher risk of TG appearance in de novo DSA patient group [22]. Due to the low number of recipients susceptible to develop TG, the possibility to lead a prospective randomized controlled trial is still unexpected. The high sensitized living kidney transplantation should be a good model to analyse the risk of DSA and TG.

The presence or absence of C4d staining deposition in TG biopsies specimen correlates poorly with the presence of HLA antibodies [26]. In a recent study, 31/48 AMR associated with TG was C4d negative and the AMR was due to anti-HLA class I and/or II in the same proportion [21]. Even if TG occurred via either HLA class I antibodies or HLA class II antibodies, 5-year graft survival in a large series of living donor kidneys with positive cross match decreased widely in class II sensitized patients as compared to that of class I sensitized patients (85.3% versus 62.6%) [4]. The presence of TG one year after transplantation results in graft loss in 30% of anti-HLA class II patients and approximately 20% of those with anti-HLA class I antibodies [4]. These authors also demonstrated a higher rate of chronic glomerulopathy (defined by a cg score > 0), which is frequently associated with chronic antibody mediated injury in the anti-HLA class II patient group [4]. The reason explaining the greater effect of anti-class II antibody as compared to class I antibody in AMR and also in TG remains unknown.

Anti-DP antibodies have been reported in TG as an only immunological cause in one patient presenting with TG [42]. Anti-DQ DSA are increasingly recognized as the predominant HLA class II DSA produced [43]. A recent study showed the clinical relevance of anti-DQ antibodies in kidney graft outcomes and confirms the pathogenic effect in cardiac and liver transplantation [44, 45]. Patients with DQ-DSA are at a higher TG risk. Interestingly, an association of Cw-DSA in TG patients was not observed suggesting a major effect of DQ DSA [46]. Issa et al. demonstrated a strong correlation between anti-class II antibody titre and the risk of TG but did not observe differences between anti-DR or anti-DQ subset effects [47].

Notably, as half of the biopsies performed 5 years after transplantation did not present evidence of TG, this raises the question of the crucial crosslink of HLA antibodies and thus suggests the involvement of other aetiologies'. To this end, some studies did not confirm an association between HLA antibodies and TG suggesting another possible mechanism in TG formation [15]. Examples include patients positive for HCV and those presenting with thrombotic microangiopathy [18, 48].

## 7. Non-HLA Antibody Involvement in TG 

Despite strong evidence for a correlation between HLA antibodies and TG, the implication of non-HLA antibodies has been suggested as a mechanism of glomerular damage occurring after transplantation. The percentage of TG cases in which HLA antibodies have not been identified is variable, with one large series from Canada suggesting that up to 27% of cases had no demonstrable antibody [26]. In our own series over 50% of cases of TG were associated with donor specific HLA antibodies [22]. Some of TG cases presented without anti-HLA antibodies or C4d staining [49] and recent reports demonstrated the role of non-HLA antibody. This autoreactivity has been well described in heart transplantation with antibody directed against myosin or vimentin [50]. Angiotensin II type 1 receptor (AT1R) antibody strongly correlated with AMR in kidney transplantation independently of DSA and confirming the rule of non-HLA Ab in acute graft injury [51]. In our well-characterized cohort of TG patients [22] we have recently identified AT1R antibodies in 52% of cases with biopsy proven TG and importantly a group of HLA antibody negative TG cases in whom AT1R antibodies were identified indicating for the first time that AT1R antibodies by themselves are associated with TG (Hanf et al. submitted). In pediatric cohort, recipients with antibodies to protein kinase Czeta developed rejection and increased the risk of allograft loss but TG was not described [52]. Using a protein screening microarray Dinavahi et al. showed that transplantation induced changes in antibody repertoires reactive to non-HLA Ag and isolated three possible pretransplant serum antibodies to peptidyl-propyl-isomerase-A, peroxisomal-trans-2-enioyl-coA-reductase (PECR), and serine threonine kinase 6 correlating with TG development. Notably, only PECR was confirmed by ELISA with a strong association between it and TG [53]. Moreover a recent study using the same process describes four new biomarkers predicting the future development of chronic allograft injury in pretransplant sampling: chemokine ligand 9 (MIG), interferon gamma (IFN*γ*), chemokine ligand 11 (ITAC), and Glial-derived neurotrophic factor [54]. Importantly, these recent pieces of data were derived from a retrospective cohort and thus require confirmation in large prospective studies.

## 8. Endothelial Cells and HLA Antibody: Crosstalk to Develop TG?

Binding HLA antibodies to HLA molecules may cause endothelial cell injury via the complement cascade and/or may induce endothelial cell proliferation and survival via intracellular signalling. Donor-specific anti-HLA alloantibodies initiate renal allograft rejection through complement-mediated and antibody-dependent cell-mediated cytotoxicity as previously described [55]. In a sensitized kidney allograft cohort, 68% of tested sera were found to contain complement-fixing alloreactivity. IgG1 type panel reactivity was predominant (detectable for HLA class I and II reactivity), followed by IgG3, and both were independently correlated. Complement fixation was also favored by the simultaneous presence of alloreactive IgG1, IgG3, and IgM [56]. IgG2 and IgG4 were more weakly involved in complement-fixing activity [57].

Electron microscopy studies show that the endothelium exhibits the first signs of injury, well before development of TG [8]. The concept of endothelial injury is supported by EC gene analysis showing a strong correlation of high expression of endothelial cell-associated transcript (ENDAT) and the presence of DSA correlating with late antibody mediated rejection. In 2009, Sis et al. described that more than 10 ENDAT genes increased ABMR predicting late graft loss. Importantly, patients presenting with DSA without high expression of ENDAT had better graft outcomes [57]. However, high ENDAT expression was also seen in borderline TG changes, T cell rejection, or polyomavirus infection [58]. Thus this marker should be interpreted in association with the presence of DSA to improve sensitivity [59]. These data suggest the correlation between HLA antibodies and EC injury mediated by complement dependent or independent pathways in chronic ABMR.

Previous studies showed that binding antibodies to class I molecules on the surface of endothelial cells results in tyrosine phosphorylation of various intracellular proteins (AKT, ERK…). The two major consequences of class I-mediated phosphorylation are cell proliferation via upregulation of fibroblast growth factor receptors (FGFR) on the surface of endothelial cells and cell survival stimulation by increased endothelial cells expression of antiapoptotic proteins Bcl-2 and Bcl-xL via the P13K/Akt pathway [60, 61]. These observations raise an important question—what are the factors that determine the outcomes of HLA class I antibody-mediated phosphorylation?

**Figure 2:**
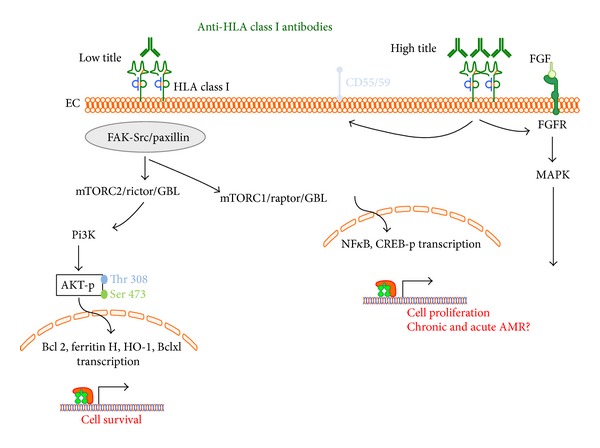
Intracellular signalling pathways mediated by high/low titre HLA class I antibodies after endothelial binding. Putative cell survival and proliferation pathways are illustrated.

As illustrated in Figure 2, studies by the Reed's group showed that stimulation of endothelial cell proliferation was observed at concentrations of HLA antibodies ranging from 0.1–10 *μ*g/mL with maximal cell proliferation at concentrations of 10 *μ*g/mL. On the other hand, treatment of endothelial cells with HLA class I antibodies for 24 hours induced a prominent increase in the prosurvival proteins Bcl-2 and Bcl-xL protein levels; with maximum increases observed with lower antibody concentrations antibodies were used (0.01–1 *μ*g/mL) [61]. These findings suggest that concentration of HLA antibodies is one of the factors contributing to the outcomes of HLA class I antibodies-mediated phosphorylation and gene expression. Furthermore, Iwasaki et al. showed that low dose HLA class I antibody activates the AKT pathway leading to the induction of antiapoptotic genes (Bcl-2) as well as the cytoprotective genes HO-1 and ferritin H [62] (Figure 2).

**Figure 3:**
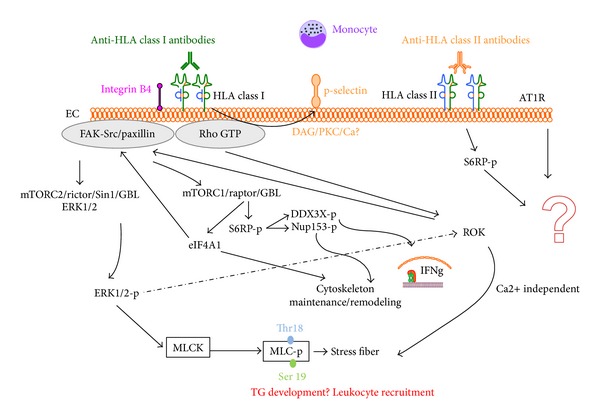
Intracellular signalling pathways mediated by HLA class I or II antibody binding to endothelial cells. The observation that the main HLA class I antibody binding stimulates intracellular EC pathways via the integrin *β*4 subunit is summarized in this figure. After stimulation cytoskeleton remodelling and stress fiber formation via ERK pathway occur with calcium dependent and independent signalling leading to possible TG development and inflammatory cell recruitment.

Recent* in vitro* work using human aortic EC addressed the question of EC cytoskeleton and antibody mediated rejection or transplant vasculopathy. In 2012 Zhang and Reed demonstrated a mutual dependency between HLA I and integrin subunit *β*4 to stimulate signal transduction and EC proliferation [63]. Ziegler et al. improved the understanding on HLA class I ligation EC pathway using both mass spectrometry analysis on cytoskeleton structure [64] as well as analysis on stress fiber formation [65]. As depicted in Figure 3, results suggest a major contribution of ERK and MLC phosphorylation in a calcium independent manner and that the remodelling in EC structure may be involved in chronic allograft rejection. Monocyte infiltration occurs during graft injury and may also contribute to the risk of TG. An elegant study by Valenzuela et al. showed,* in vitro* and* in vivo*, a role for p-selectin in monocyte recruitment induced by HLA class I Ab [66] (Figure 3). Taken together, these results confirm the corner stone role of HLA antibodies in the chronic rejection and thus warrant further investigation in TG formation.

Interestingly, the majority of studies investigating antibody activity in EC injury were mediated by HLA class I monoclonal antibodies leaving clear need to investigate class II antibodies. One study investigated the effects of class I and II antibodies on EC on cardiac allografts and showed that phosphorylation of S6 ribosomal protein (S6RP), a downstream target of the PI3K/Akt/mTOR pathway, was a biomarker of antibody mediated rejection [67]. The authors demonstrated that ligation of HLA class I and class II molecules on EC resulted in increased phosphorylation of S6RP. Then they showed in a cardiac biopsy that antibody production to class II antigens was positively associated with p-S6RP-positive staining and confirmed a strong association between generation of DSA to class II antigens and EC staining of p-S6RP staining. These observations were not significant with class I Ab or DSA. These data suggest crosstalk between class II Ab and EC but at present there is a lack of* in vitro* or animal studies with class II DSA or class II antibodies. Le Bas-Bernardet et al. showed that DR expression was sufficient to trigger intracellular signaling in EC isolated from human deceased donor, in response to HLA-DR ligation. Crosslinking of HLA-DR on ECs promotes Akt activation and phosphorylation, suggesting that the PI3-K pathway, involved in EC survival, was activated. These two studies on class II antibodies raise the question of survival signalling pathways contributing to EC changes. However the EC used (from human large vessels) are quite different from those of glomerular EC likely specifically involved in the TG process [68, 69]. HLA antibodies tested in glomerular EC subset demonstrated that these were able to produce complement component (C3 and C4) [70].

Non-human primate studies in cynomolgus monkeys have further added to our knowledge of the pathogenesis of TG suggesting that there are 4 stages of the process. The initiating stage is increased donor-specific antibodies, followed by C4d deposition, then development of tissue injury, and finally decrease in allograft function [71].

## 9. Treatment Options

Interestingly, the current recommendations in TG are not based on randomized controlled trials or level 1 evidence but rather on expert advice. Moreover, there is no efficient treatment to limit TG progression with treatment based on preventive recommendations (e.g., monitoring DSA, avoiding and controlling antibody mediated rejection, and reinforcing medication compliance) [72, 73]. The use of antiproteinuric agents (e.g., ACE and ARB) is currently ongoing [74]. Different desensitization protocols have been used in sensitized patients at risk for antibody mediated rejection [73, 74] and transplantation teams replayed these different strategies in chronic ABMR and/or TG without major significance benefits [12].

The diagnosis of TG in early stage should probably be a goal but it requires systematic biopsies with electron microscopy to dissect endothelial change. Thus it should be recommended for electron microscopy to be performed on all transplant biopsies in patients at risk of developing TG to enable early detection of these changes. In line with the recent consensus guideline on the testing and clinical management issues associated with HLA and non-HLA antibodies in transplantation protocol biopsies should be performed once de novo DSA have been detected [38]. A recent study showed a benefit in the rate of development of TG in patients presenting glomerular ultrastructural changes and DSA and receiving IG IV + plasmapheresis and/or rituximab [15]. However a pilot study using rituximab did not show efficacy in TG with stabilisation of TG in 50% of the cases [48] but these results and other strategies should be performed in prospective trials with TG and DSA monitoring as key end points in the trial design [38]. The use of rituximab or splenectomy in incompatible ABO transplantation was efficient to treat chronic ABMR in ABO incompatible kidney transplantation with reduction of DSA titre [74]. Lefaucheur et al. suggested that using plasma exchange in association with high dose of intravenous immunoglobulin's and rituximab reduced DSA level and AMR three years after transplantation and thus reducing the risk of chronic AMR [75].

Based on B cells depletion efficacy in experimental models [76, 77], bortezomib showed an efficacy in chronic AMR case studies [78] and in AMR treatment [79]. A controlled trial is ongoing to investigate if bortezomib will prevent TG in patients who are at high risk of developing the condition due to high donor-specific alloantibody in posttransplant kidney recipients (NCT01349595 on ClinicalTrials.gov).

The other new drug in the field is the C5 inhibitor eculizumab (anti-C5 humanized monoclonal antibody; Alexion, Cheshire, Connecticut). This agent was initially developed for paroxysmal nocturnal hemoglobinuria, the implication of complement pathway in AMR lead transplantation team, to test eculizumab in sensitized patients with excellent results [80]. It is also suggested that one of the pathways resulting in TG is complement dependent due to the presence of C4d deposition in biopsies. Stegall et al. showed that sensitized patients receiving eculizumab decreased the rate of AMR [81]. Even if the protocol biopsy at one year after transplantation tends to show efficacy in TG development, the same group present, during the American Transplantation Congress 2012, results until 2 years after transplantation from positive crossmatch patients receiving eculizumab. They demonstrated similar rate of TG in both groups at two years posttransplantation (50% versus 55% in eculizumab versus control group, resp.) [82]. These results suggest that some case of early TG may involve with independent complement pathway.

## 10. Conclusion

TG remains a major cause of graft loss. We described a strong correlation between EC injuries and HLA antibody likely involved in TG process. The recent knowledge in intracellular pathways involved in transplant vasculopathy after HLA antibody ligation to their receptor in EC will likely improve in the future years and we hope that could be extend to the concept of TG to develop new TG formation blockade strategies. Treatment recommendations are mainly preventive because treatment targeting HLA Ab or their consequences did not shown encouraging results, especially for eculizumab. The main and only prospective study devoted to TG is ongoing and should be an alternative to the current therapy based on HLA Ab removal.

The unraised question about allo- or auto-non-HLA antibody and TG remains open and warrants address to determine the contribution of these antibodies in TG as well as the HCV status and thrombotic microangiopathy disease.

## Figures and Tables

**Table 1:** Studies including TG patients a/reporting different class I or II antibodies anti HLA a/DSA are expressed on percentage.

	Year	Number of TG patients	Time to biopsy diagnosis	Proteinuria	Anti-HLA Class I	Anti-HLA Class II	Both	Class I DSA	Class II DSA	Both DSA	Antibody detection method
Sun et al. [6]	2012	43	4.53 years	2.01 g/24 h	30	56.7		/	/	/	FlowPRA
Wavamunno et al. [8]	2007	7	2.3 years (LM)	70%	/	/	/	/	/	71.4	Luminex SA
Sijpkens et al. [9]	2004	18	8.3 years	3.1 g/24 h	/	/	/	7.6	23	7.6	ELISA
Haas and Mirocha [15]	2011	8	10.1 months	/	/	/	/	13	38	50	Luminex SA
Gloor et al. [19]	2007	55	21 months	1.48 g/24 h	/	/	76	7	22	46	Luminex SA
Eng et al. [22]	2011	61	4 years	/	54	32	/	53	47	58	Luminex SA
Lopez Jimenez et al. [25]	2012	30	7.1 years	1.9 g/24 h	/	/	40	/	/	23.3	/
Sis et al. [26]	2007	41	5.5 years	Dipstick 26+	6.3	27.6	36.2	12.1	42.4	30.3	FlowPRA
Rostaing et al. [48]	2009	14	9.3 years	2.35 g/24 h	/	/	/	7	36	43	ELISA Luminex ND

SA: single antigen; ND: not defined.
